# Counting cells in motion by quantitative real-time magnetic particle imaging

**DOI:** 10.1038/s41598-024-54784-5

**Published:** 2024-02-21

**Authors:** Amani Remmo, Olaf Kosch, Lena Kampen, Antje Ludwig, Frank Wiekhorst, Norbert Löwa

**Affiliations:** 1 Physikalisch-Technische Bundesanstalt, Abbestr. 2-12, 10587 Berlin, Germany; 2 Department of Cardiology, Angiology and Intensive Care Medicine, Deutsches Herzzentrum der Charité, Charitéplatz 1, 10117 Berlin, Germany; 3 Charité – Universitätsmedizin Berlin, Corporate Member of Freie Universität Berlin and Humboldt-Universitätzu Berlin, Charitéplatz 1, 10117 Berlin, Germany; 4 DZHK (German Centre for Cardiovascular Research), Partner Site Berlin, Berlin, Germany

**Keywords:** Cell biology, Molecular biology, Biomarkers, Medical research, Nanoscience and technology, Nanomedicine

## Abstract

Magnetic Particle Imaging (MPI) is an advanced and powerful imaging modality for visualization and quantitative real-time detection of magnetic nanoparticles (MNPs). This opens the possibility of tracking cells in vivo once they have been loaded by MNPs. Imaging modalities such as optical imaging, X-ray computed tomography (CT), positron emission tomography (PET), single photon emission computed tomography (SPECT), and magnetic resonance imaging (MRI) face limitations, from depth of penetration and radiation exposure to resolution and quantification accuracy. MPI addresses these challenges, enabling radiation-free tracking of MNP-loaded cells with precise quantification. However, the real-time tracking of MNP-loaded cells with MPI has not been demonstrated yet. This study establishes real-time quantitative tracking of MNP-loaded cells. Therefore, THP-1 monocytes were loaded with three different MNP systems, including the MPI gold standard Resovist and Synomag. The real-time MPI experiments reveal different MPI resolution behaviors of the three MNP systems after cellular uptake. Real-time quantitative imaging was achieved by time-resolved cell number determination and comparison with the number of inserted cells. About 95% of the inserted cells were successfully tracked in a controlled phantom environment. These results underline the potential of MPI for real-time investigation of cell migration and interaction with tissue in vivo.

## Introduction

Magnetic Particle Imaging (MPI) is an advanced imaging technique that provides high temporal resolution in visualizing the spatial distribution of magnetic nanoparticles (MNPs). MPI holds great promises in enhancing medical diagnostics by enabling the real-time tracking of intravenously administered MNPs throughout the body^1,2^. This capability facilitates a deeper understanding of organ function^3^, tissue perfusion^4^, detection of bleeding sources^5^, and diagnosis of brain-related diseases such as stroke^6^. MNPs, typically coated with biocompatible polymers, play a pivotal role in the success of MPI. The design of these particles aims to reduce early clearance from the body^7,8^, ensuring sustained imaging capabilities.

An innovative strategy involves pre-introducing them into cells before injection^9,10^, effectively prolonging their circulation time, detecting inflammatory processes^11^, or overcoming biological barriers, such as the blood brain barrier^12,13^. This approach utilizes MPI for non-invasive visualization of cell fate through cell tracking. Cell tracking involves monitoring individual cells or cell groups over time, offering potential applications in the early detection of diseases, particularly in the study of inflammatory processes^14^. MNP-labeled THP-1 monocytes^15,16^, a type of white blood cell derived from human leukemia patients are a promising tool for medical diagnostics due to their remarkable ability to detect and respond to inflammation^17^.

Various imaging modalities have been explored for in vivo, non-invasive cell tracking, including optical imaging, X-ray CT, PET, SPECT, and MRI. However, each of these approaches comes with its own set of limitations. Optical imaging faces challenges in terms of penetration depth, quantification accuracy, and resolution^18^, while CT raises concerns about tracer loading impacting cell viability and function^19–21^. PET and SPECT are hindered by radiation exposure, short-lived tracers, and limited resolution. MRI, though radiation-free, becomes challenging in achieving quantitative cell tracking due to indirect imaging of MNPs^22–24^. Thus, achieving quantitative cell tracking with established imaging modalities remains a persistent challenge. Our goal is to enhance in vivo cell tracking in biomedical research by utilizing MPI, thereby achieving greater precision, speed, and quantitative results^14,25,26^. The MPI signal has been proven to correlate linearly with both the MNP concentration and the number of MNP-loaded cells, enabling precise cell quantification with high sensitivity^27^. Through tracer bolus injection studies utilizing an innovative MPI surface coil designed for mice, Graeser et al. estimated the remarkable detection limit of fewer than ten MNP-loaded cells, showcasing the potential for detecting even individual cells with MPI^28^. Therefore, a reproducible and controlled procedure for labelling cells with MNPs for MPI is necessary. Various factors, including incubation time, culture medium conditions, and the structural properties of MNPs, can affect the internalization of MNPs by cells. When loading cells with MNPs for MPI, it must be considered that the magnetic properties of the MNPs, and thus the quality of the MPI signal, may be altered^29–32^. However, these changes could not be attributed to changes in the MNP microstructure and the environment. Only the magnetic interactions between the MNP proved to be the main cause of the reduced magnetic performance in cells^30^. While cell labeling with MNPs seems to be complex, MPI has already proven its utility in various applications for visualizing the in vivo biodistribution of magnetically labeled cells over extended periods, spanning hours and days^33–38^. While the potential of MPI for real-time cell imaging is widely recognized, exploiting its capabilities, particularly in capturing the dynamic motion of cells with millisecond temporal resolution, remains an unexplored area. The application of MPI to gain quantitative real-time insights into cell dynamics, particularly in motion, has yet to be demonstrated. This untapped potential underscores the need for further research and development to capitalize on MPI's unique properties and deliver on its promise for unmatched temporal precision in cell imaging. The aim of this study was to establish real-time quantitative tracking of MNP-loaded cells in motion. We illustrated this by employing the human myelomonocytic cell line THP-1 using MPI. Moreover, the impact of the MNP material on the accuracy of quantitatively tracking cells was investigated. Therefore, the cells were loaded with three different MNP types. Apart from the MPI gold standard Resovist, we employed two MNP systems of the Synomag series, each with excellent MPI performance and special coatings^39^. After evaluating loading status and MPI performance of the MNP-loaded cells, we investigated their distinct behaviors in a real-time bolus experiment using a pre-clinical MPI scanner. Validation of quantitative imaging was achieved through time-resolved cell number determination and subsequent comparison with the number of inserted cells.

## Material and methods

### Magnetic nanoparticles (MNPs)

We used commercially available and well-established MNP systems. Two different MNP systems from the manufacturer *Micromod Partikeltechnologie GmbH* (Rostock, GER) are investigated. The first MNP system is plain Synomag before coating and functionalization (SynP50) with a nominal hydrodynamic size (total size, e.g., shell plus core diameter) of 50 nm. And the second MNP system from *Micromod* is citrate coated Synomag with a nominal hydrodynamic size of 30 nm (SynC30). Additionally, Resovist (*Bayer Pharma AG*, Berlin, GER) known as the MPI gold standard with a nominal hydrodynamic size of 60 nm^40^ is used in this work. For detailed insights into the nanoscale morphology and distribution of the MNPs, Transmission Electron Microscopy (TEM) images are presented in Appendix A.1. Regrettably, no TEM images are available for SynP50.

### Media

All chemicals used in this study were of analytical reagent grade. As media deionized water (ddH2O), phosphate-buffered saline (PBS, *Gibco*, GER), fetal calf serum (FCS, *Biochrom*, GER), and Roswell Park Memorial Institute 1640 medium (RPMI 1640 Medium, *Gibco*, GER) have been used.

### Cell line: THP-1 monocytes

The cell line THP-1 (AmericanType Culture Collection ATCC TIB202), derived from a patient with acute monocytic leukemia, was purchased from *LGC Standards GmbH* (Wesel, GER). The cells were cultured in cell culture flasks using RPMI medium 1640 (*Gibco*, GER) in an incubator set to 37 °C at a 5% CO_2_ atmosphere. The culture medium was supplemented with 10% fetal calf serum (FCS, *Biochrom*, GER) and 1% penicillin–streptomycin (*Invitrogen*, GER). The culture medium was refreshed every 2–3 d. Once the cell density reached 5·10^5^ cells/mL, the suspended THP-1 monocytes were diluted with fresh medium at a ratio of 1:3. 5·10^5^ cells were incubated with MNPs at an iron concentration of *c*_Fe_ = 0.5 mmol/L at 37 °C. After eight minutes of incubation, the cells were centrifuged at 800 rpm for 2 min. The cell pellet was washed once with PBS and resuspended in 500 µL of 0.05% glutaraldehyde (*Sigma Aldrich*, GER). Cells were transferred to an ibidi 4-well µ-slides (*ibidi GmbH*, GER) and fixed for 45 min at room temperature. For visual inspection of MNP-loaded cells, light microscopy images were made using a microscope (Axio Observer 7 ACR, *Carl Zeiss Microscopy,* GER). For microscopy, the cells were fixed to maintain a stable state and preserve their structural characteristics. While fixing, cells are simultaneously preserved and terminated by the addition of glutaraldehyde, maintaining their current state. The glutaraldehyde was removed from the top and 500 µL of a 1:1 solution of 2% hydrochloride acid (*Merck,* GER) and 4% potassium ferrocyanide (*Sigma-Aldrich*, GER) was added. After 10 min of incubation at room temperature, Nuclear Fast Red (*Carl Roth*, GER) was added to visualize the cell nuclei. After 10 min of incubation at room temperature, cells were visualized in the microscope (63 × objective). The Z-stacks technique was employed to capture cells in various dimensions. For this, the microscope's focal plane is incrementally adjusted along the z-axis while acquiring images. The separation between consecutive planes is referred to as the Z-step. In this instance, the Z-step was 0.28 µm.

All cell samples were labeled with MNPs by exposure to an MNP suspension containing 28 µg of iron (*V* = 1000 µL, *c*_Fe_ = 0.5 mmol/L), followed by the removal of non-absorbed MNPs through a washing procedure as previously described^17^.

### Spectrophotometric determination of iron in cells

For spectrophotometric determination of iron in MNP-loaded cells, the 1,10-phenanthroline protocol was used. Pellets of MNP-loaded cells were first dissolved in 37 % hydrochloric acid (HCl, *Carl Roth*, GER). Subsequently, aliquots of the solution were mixed with hydroxylamine hydrochloride, 1,10-phenanthroline hydrochloride, acetic acid, and sodium acetate. This results in the formation of a colored complex ion as iron(II) reacts with 1,10-phenanthroline^16^. The concentration of iron is then inferred by measuring the absorbance at 495 nm of the solution using a photometer (SpectraMAX Plus 384, *Molecular Devices, UK*). A calibration curve was established to correlate absorbance with iron(II) concentration, enabling the determination of the concentration of an unknown sample.

### Magnetic particle spectroscopy (MPS)

MPS measurements were conducted on a commercial device (MPS-3, *Bruker*, GER) operated at an excitation amplitude of *B*_ex_ = 12 mT, and a frequency of *f*_0_ = 25 kHz. This device detects the non-linear dynamic magnetic response of MNPs exposed to the alternating magnetic field. For the measurement a fast reaction tube (PCR tube, *Applied Biosystems,* US) containing a sample is placed into the detection coil of the MPS detecting the induced dynamic magnetization of the MNPs in the sample. By Fourier transformation of the time signal the spectral components are obtained showing distinctive amplitudes at odd multiples (harmonics) of the excitation frequency *f*_0_. As shown previously^41^, the MPI performance of a MNP system can be characterized from related MPS measurements using the characteristic parameter *k*_LOD_ that is the number of MPS harmonics* A*_k_ above the limit of detection (LOD) of the MPS device. The limit of detection (LOD) of the MPS device was determined according to the guidance of the International Union of Pure and Applied Chemistry (IUPAC): LOD (*A*_k_) = *µ*_k_ + 3·*Ϭ*_k_ of ten empty-sample-holder (background) measurements, where *µ*_k_ is the mean of *A*_k_, and σ_k_ is the standard deviation from ten empty sample holder measurements.

### Magnetic particle imaging (MPI)

MPI measurements were conducted using a commercially available preclinical MPI scanner (*Bruker* MPI 25/50 FF, GER) that generates a field-free-point (FFP) with a static magnetic field gradient (1.25/1.25/2.5 T/m in x/y/z-direction). The MPI scanner is equipped with an additional separate mouse coil (*Bruke, GER*) with an inner diameter of 44 mm to increase the sensitivity. By superimposing oscillating magnetic drive fields with field amplitudes of 12 mT at slightly different frequencies (24.5/26.0/25.3 kHz in x/y/z-direction) the FFP is moved on a Lissajous trajectory covering a field of view (FoV) of 19.2 × 19.2 × 9.6 mm^3^. A gradiometric receive coil measured the time signal from which the harmonic spectrum was derived by Fourier transformation. The spectral pattern of the MPI signal ($\overline{u }$) depends on the MNP system, the environmental conditions, and the spatial distribution of the MNP within the FoV. A three-dimensional image reconstruction of the MNP distribution is performed by solving the following inverse problem by least-squares methods using the *Kaczmarz* algorithm with *Tikhonov* regularization (regularization parameter $\lambda$): ${\Vert \overline{{\text{S}} }c-\overline{u}\Vert }^{2}+\lambda {\Vert c\Vert }^{2}\to \mathrm{ min}$.

#### System function (SF)

The SF ($\overline{{\text{S}} }$) acquisition can be considered as a calibration procedure measuring the MPI signal $\overline{u }$ of a point-like MNP system (*V*_ref_ = 27 µL, 3 × 3 × 3mm^3^) at each spatial position in the FoV. The SFs were recorded using 100 averages subtracted by a background measurement. The SF for the three MNP system has been recorded in a volume of 25 × 25 × 13 voxel, with a voxel size of 1 × 1 × 1 mm^3^. The SF acquisition took 9 h. The same settings as for the SFs were used for an empty scanner measurement. For the evaluation of the real-time quantitative cell tracking, the amount of MNPs in the cell samples was determined relative to the amount of the reference sample that was used for the SF acquisition. For the reference sample 5·10^6^ MNP-loaded cells (10 min incubation) were used for SF acquisition instead of using a reference sample prepared directly from MNP stock suspension.

#### Phantom setup for real-time, quantitative MPI cell tracking

**Figure 1:**
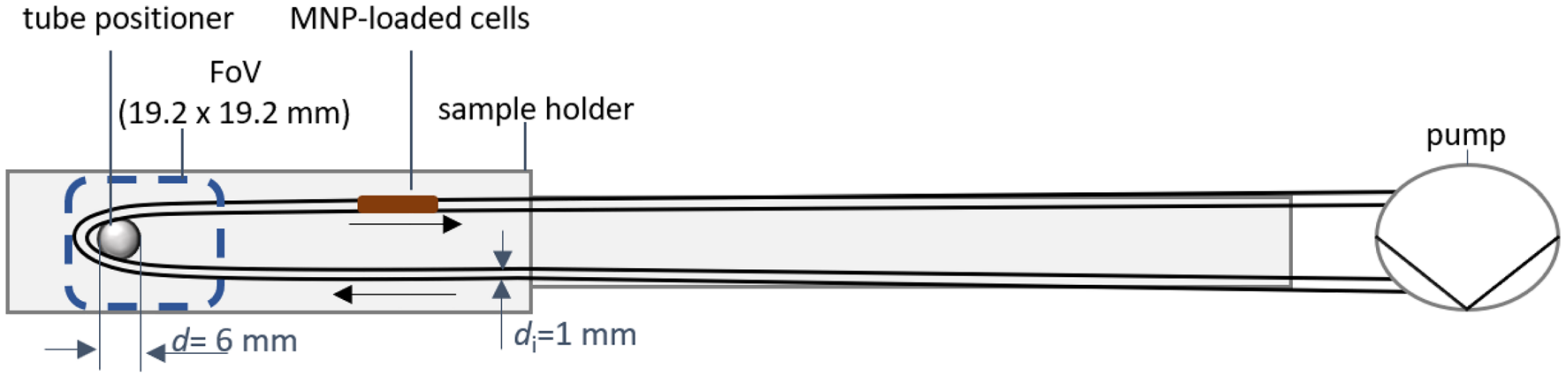
Experimental setup for real-time cell tracking using a MPI scanner. MNP-loaded cells were located in a tubing and moved through the FoV (blue dashed line) using a high-pressure multichannel pump (Ismatec). The tube (inner diameter *d*_i_ = 1 mm, length *l* = 1 m.) was attached to an MPI sample holder and inserted into the mouse coil of the MPI scanner.

To facilitate real-time cell tracking, a volume of *V* = 100 µL containing 5∙10^6^ cells in PBS was introduced into a tube (material: polyvinyl chloride (PVC), inner diameter *d*_i_ = 1 mm, length *l* = 1 m). Silicone oil was employed as a carrier medium within the tube to prevent MNP-loaded cells from adhering to tube walls. The tube was bent 180° within the FOV of the MPI scanner, positioning 0.026 mL inside the FoV. (see Fig. 1). The MNP-loaded cells are carried within the liquid flow through the tube at a flow rate of 0.5 mL/min. This was performed individually for each of the three distinct MNP-labeled cell types (SynP50, SynC30, and RES). The measurements were recorded with a temporal resolution of 21.5 ms. A background signal (duration *t* = 6.45 s), recorded prior to the measurement, was subtracted. In this study, the full temporal resolution of MPI was not exploited. By tenfold averaging—resulting in a temporal resolution of 215 ms—the transport of cells could be fully resolved. Due to the resulting reduction of noise, a lower regularization could be used in the reconstructions. The *Kaczmarz* algorithm with *Tikhonov* regularization was applied for 3d reconstruction with 20 iterations and a regularization factor λ_r_ = 2 $\cdot$ 10^–2^ for the cells containing SynP50, and SynC30. A higher regularization factor (*λ*_r_ = 50) was necessary for the RES-containing cells owing to their weak MPI signals. All frequency components with SNR ≥ 4 were included into image reconstructions. The number of frequency components is equivalent to the number of equations used in the reconstruction when solving the system of equations: $\overline{S }\cdot c=\overline{u }$. The higher the number of equations above the noise level, the more geometric detail can be reconstructed, which in principle leads to an increase in resolution. For the quantitative evaluation of the cell number *N*_cells_ in each voxel, a reference sample with known cell number (*N*_cells_ = 5 $\cdot$ 10^6^) was used for the SF acquisition. To quantify cells within the FoV over time, the contributions of all voxels with a content greater than zero were summed up.

## Results and discussion

### Labeling of THP-1 monocytes for MPI

**Figure 2:**
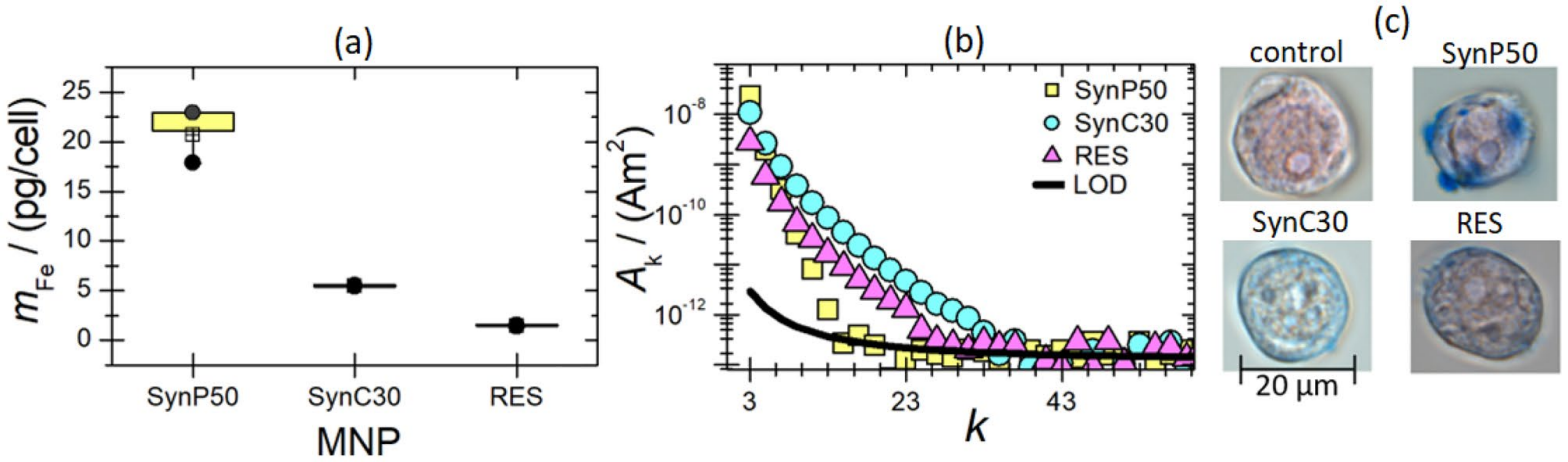
(**a**) Box plots of the internalized MNP amounts (iron mass/per cell) of SynP50, SynC30, and RES after an incubation time of 10 min. An iron mass of 28 µg was added to the cells (V = 1000 µL, c_Fe_ = 0.5 mmol/L). The uncertainty was calculated based on three repeated measurements and corresponds to the standard deviation (coverage factor *k *= 1). Note that for SynC30 and RES the individual three replicates are so close that they coincide within the symbol thickness. (**b**) Amplitude spectra *A*_k_ of SynC30, SynP50, and RES after cellular uptake (10 min incubation, exposed iron mass of 28 µg) and washing of the MNP-loaded cells. (**c**) Microscopy images of the cells (scale bar = 20 µm) incubated with SynP50, SynC30, and RES. The MNP and MNP aggregates were stained with Prussian blue, while the cell nucleus was stained with Nuclear Fast Red. Here, the cells were washed once. The first microscope image shows a control image of the cells before cellular uptake.

In MPI cell tracking, a crucial aspect is the MNP loading state of the cells, e.g., the quantity of internalized MNP per cell. Figure 2a presents the average internalized iron amount per cell after a 10-min incubation time for the three MNP systems (SynP50, SynC30, and RES).

The highest cellular uptake of 20(3) pg/cell is measured for SynP50, which corresponds to 71% of the applied MNP amount. SynP50 represents the Synomag MNP core before the coating and functionalization process. Synomag with a citrate coating, SynC30, shows a reduced cellular uptake of 5(1) pg/cell, which corresponds to 18% of the applied MNP amount. Dextran-COOH coated RES exhibits a very low cellular uptake of 1.5(2) pg/cell that corresponds to 5% of the applied MNP amount.

In addition, to quantify the internalized MNP amount per cell, it is crucial to conduct magnetic characterization of MNPs after cellular uptake to ensure a reliable assessment of the dynamic magnetic properties of MNPs within cells. This is accomplished by MPS measurements of cell samples after MNP uptake, which reveal significant differences in the amplitude spectra *A*_k_ for the three MNP systems (Fig. 2b). Though MNP-loaded cells with the highest iron content (SynP50) exhibit the highest *A*_3_ signal amplitude, the amplitude spectrum is notably steep, resulting in a low number of MPS harmonics* A*_k_ above the LOD of *k*_LOD_ = 15. Despite the limited cellular uptake of RES with its rather flat amplitude spectrum, the *k*_LOD_ value of 27 is rather large. SynC30-loaded cells exhibit a moderate *A*_3_ signal amplitude and a relatively flat spectrum that yields a *k*_LOD_ = 33. It should be considered that the signal behavior of all MNPs significantly differed in the initial state and in cells. While the spectrum of RES and SynP50 became significantly steeper in cells, it has, interestingly, become flatter in the case of SynC30 (see supplementary material, Figure A.2). The microscope images (see Fig. 2c) clearly show the different cellular uptake behavior of SynC30, SynP50, and RES. While SynC30, and RES are visibly internalized by cells, SynP50 is subjected to a notable adsorption at the cell surface. It is known that up to a certain size, objetcs can be taken up by monocytes via macropinocytosis and phagocytosis^42,43^. It could be possible that SynP50 aggregates exceed this size and explaining the observed attachment to the outside of cells. Therefore, SynP50 is unsuitable for cell tracking because SynP50 particles on the surface could easily detach during their way inside a body and thus generate a false positive signal in MPI imaging.

Note that all cell samples underwent a special washing procedure before and after incubation with MNP according to^29^.

### MPI performance of MNP-loaded cells

To assess the spatial resolution of the three MNP systems SynP50, SynC30, and RES in cells, we conducted the two-voxel analysis as described previously^44^. Instead of using a reference sample prepared directly from MNP stock suspension, 5·10^6^ MNP-loaded cells were used for SF acquisition.

**Figure 3:**
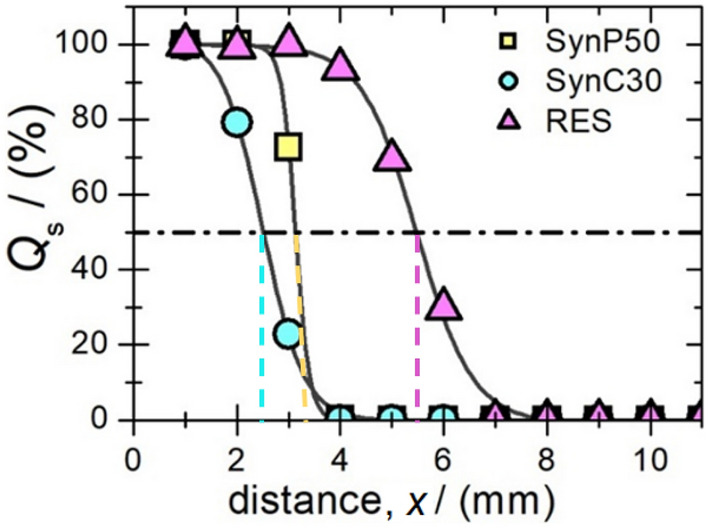
MPI spatial resolution determined by the SF-based two-voxel analysis using MNP-loaded cells. The separation quality Q_S_ as a function of distance (number of empty voxels) for SynP50 (yellow squares), SynC30 (blue spheres), and RES (magenta triangles) incubated with 5 $\cdot$ 10^6^ cells is shown and fitted with a Heaviside function (black line) to determine the resolution r that is defined as the voxel distance x at which the separation quality *Q* is 50% (dashed line).

Figure 3 illustrates the separation quality *Q*_s_ as a function of distance *x* (number of empty voxels) as obtained by the two-voxel analyzes for samples containing MNP-loaded cells from which the MPI resolution *r* is extracted^44^. The resolution *r* is defined as the voxel distance *x* at which *Q*_s_ is 50%. The best resolution of *r* = 2.50(8) mm is determined for SynC30. For SynP50-loaded cells, a resolution of *r* = 3.31(5) mm is extracted. For RES a resolution *r* above 5 mm is determined. However, these results consider the noise contribution from a real cell tracking measurement since the number of FC is determined from SF data and noise measurement. Furthermore, the final image quality depends on how well the SF matches the characteristics of the MNP-loaded cells in the measured experimental setting. By combining the findings from section "Labeling of THP-1 monocytes for MPI", where we determined the absorbed iron mass and the magnetic properties of MNP-loaded cells, and the evaluation of spatial resolution through two-voxel analysis, an intriguing observation arises: there isn't always a direct correlation between high iron uptake and optimal spatial resolution in MPI. This observation is evident when comparing SynC30 and SynP50. SynC30, despite having three times less internalized iron compared to SynP50, achieves superior spatial resolution in MPI owing to its larger number of harmonics above the noise floor (SynC30 *k*_LOD_ = 33, SynP50* k*_LOD_ = 15). Despite RES generating twice as many harmonics compared to SynP50 (*k*_LOD_ = 27), it exhibits inferior MPI performance (resolution), which may be attributed to a tenfold lower iron uptake.

### Real-time MPI tracking of MNP-loaded cells

**Figure 4:**
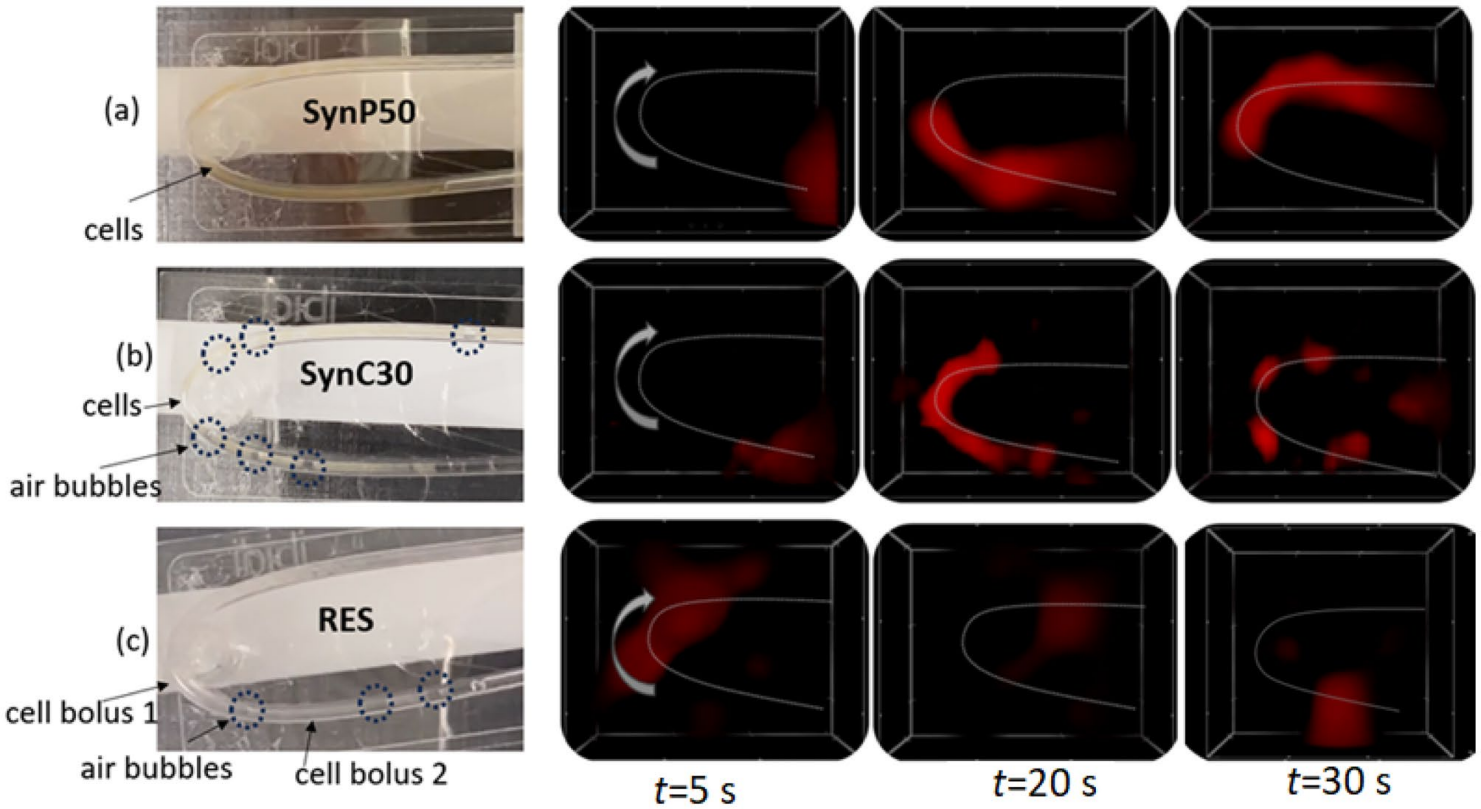
Images of the experimental setup showcasing the tube phantom filled with MNP-loaded cells positioned on the MPI sample holder (white box). Followed by MPI frames of the real-time flow measurements of cells loaded with (**a**) SynP50, (**b**) SynC30, and (**c**) RES at the time points *t *= 5 s, 20 s, and 30 s after injection. The dashed line represents the path of the tube.

To simulate a real MPI cell tracking scenario, we conducted real-time flow measurements using MNP-loaded cells for the three systems: SynP50, SynC30, and RES. The MNP-loaded cells were transported by a laminar flow inside a tube during MPI acquisition (see 2.6). For each of the three MNP-loaded cell systems, Fig. 4 displays images of the arrangement of MNP-loaded cells in the tube together with reconstructed MPI image frames of the MNP-loaded cells at time points of 5 s, 20 s, and 30 s (full time video is found in supplementary material). All three MNP-loaded cells systems could be tracked in real-time by MPI with different degrees of resolution and seemingly varying consistencies of the cell pellets. Particularly in the case of SynP50-loaded cells, where the MNP tend to adhere to the cell surface, the cell pellet maintained as a compact bolus with high density during the flow experiment (see 3.1). In cells loaded with the other two MNP systems, air bubbles in the tube formed, causing these cell pellets not to move through the tube as a compact bolus anymore but to form several hotspots that can clearly be resolved in the MPI images of SynC30-loaded cells. For RES, the MPI images reveal the appearance of an additional, second bolus after 30 s, seemingly separated from the primary bolus within the flow.

### Validation of real-time quantitative MPI cell tracking

**Figure 5:**
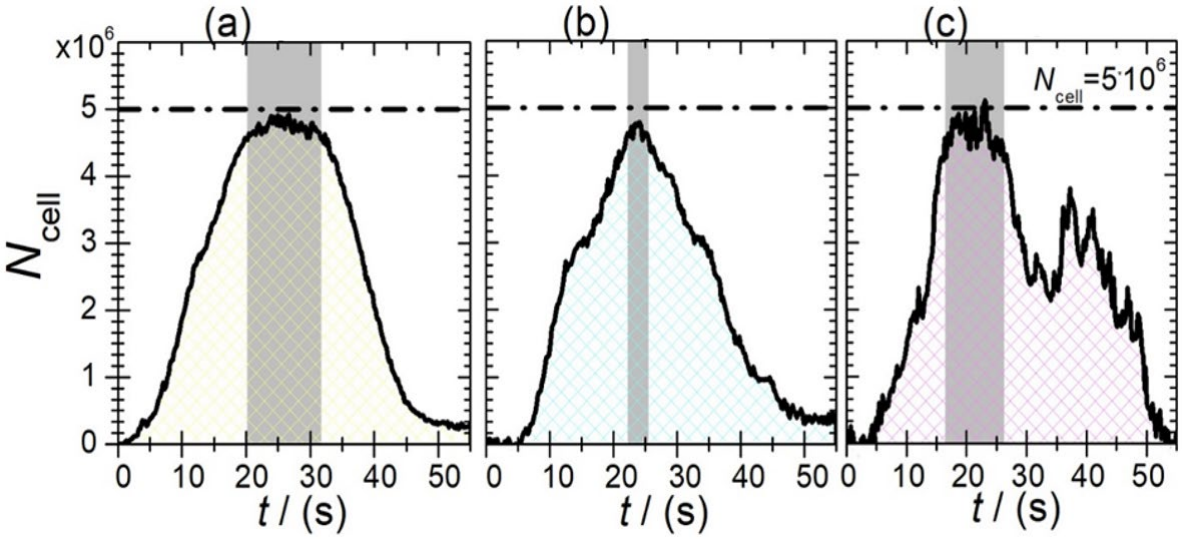
Quantitative evaluation of real-time flow measurements of MNP-loaded cells with (**a**) SynP50 (yellow), (**b**) SynC30 (blue), and (**c**) RES (magenta). The gray area shows the signal maximum reached by the MNP-loaded cells.

Figure 5 shows the quantitative MPI evaluation of the real-time flow measurement with MNP-loaded cells. Here, the quantified number of MNP-loaded cells in the FoV is provided during an acquisition time of 55 s duration. A total number of 5·10^6^ cells were used for each experiment (see dashed line in Fig. 5). For image reconstruction of SynP50 and SynC30 (Fig. 5a,b), a low regularization factor of *λ*_r_ = 2 $\cdot$ 10^–2^ was used whereas for RES this value was increased to *λ*_r_ = 50.

Quantitative analysis revealed that between 20 and 31 s (as marked by the shaded area in Fig. 5a), SynP50 a distinct maximum of loaded cells within the FoV was recovered in the images frames, with an average of 4.75(9)·10^6^ cells. This represents a 95% retrieval of the initially introduced 5·10^6^ cells. The time window for the signal peak for SynC30-loaded cells (22 s to 25 s, see Fig. 5b) is shorter compared to SynP50. During this period 4.68(9)·10^6^ cells were recovered, equivalent to 93.5% of the initially introduced 5·10^6^ cells. The time interval for the maximum number of RES-loaded cells in the FoV is 16 s to 26 s as shown in Fig. 5c. For RES (see supplementary material), the cell bolus was splitted into two parts, resulting in two peaks in the quantification (see Fig. 5). In total, 4.7(2)·10^6^ RES-loaded cells were identified, with 93.1% of the initially introduced cells being retrieved.

To estimate the minimum detectable cell quantity, the mean value of the blank signal over the entire FoV, measured 10 s before the bolus was released, was calculated and then divided by the number of voxels. The resulting mean background signal per voxel corresponded to cell numbers of 20, 83, and 86 cells per voxel for SynP50, SynC30, and RES, respectively.

As can be seen in Fig. 5, the quantified the cell numbers do not return to zero after the cell bolus has left the phantom once, which is ascribed to the partial retention of MNP-loaded cells at the tube walls.

**Figure 6:**
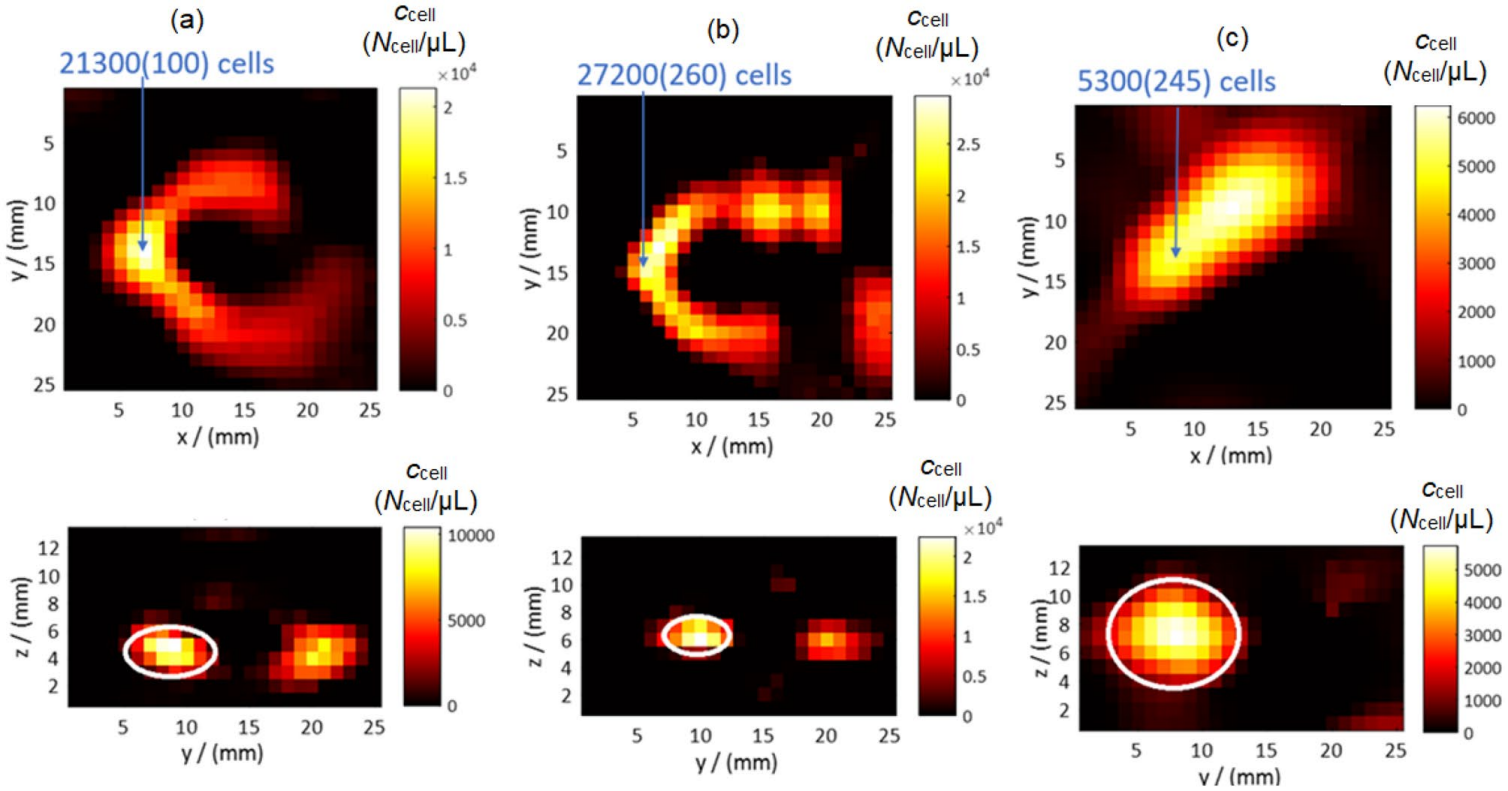
Quantitative MPI image slices during the real-time tracking of (**a**) SynP50-, (**b**) SynC30- and (**c**) RES-loaded cells at the time of maximum cell number in FoV *t*_max_ = 24.3 s. The top row shows the *x*–*y* plan, where the tube was positioned. For SynP50, and RES the 5th layer was selected, whereas for SynC30 the 6th layer is shown, which is due to slight variations in the height of the tube phantom in the FoV during each experiment. The bottom row displays the z–y plane containing the cross section of the tube to determine the diameter of the MNP-loaded tube.

In addition to the quantitative analysis described above, Fig. 6 shows representative MPI image slices for the three MNP systems with color-coded cell concentration *c*_cell_ of each voxel. The images were selected at a common time point (*t*_max_ = 24.3 s) where a maximum signal was detected for all three systems.

The reconstructed tube cross-section is elliptical, resulting from different imaging gradients in the *y*- and *z*-planes. The *z*-plane resolution is double that of the *y*-plane, shaping the tube with diameters *d*_y_ and *d*_z_ along the *y* and *z*-planes, respectively. When comparing the tube inner diameter *d*_i_ = 1 mm through which the MNP-loaded cells are transported with the reconstructed width *d*_rec_, the best agreement is found for SynC30-loaded cells (*d*_z_ ~ 2.2 mm, *d*_y_ ~ 5 mm), see Fig. 6b. The image reconstruction of SynP50-loaded cells (see Fig. 6a) appears to be more blurred (*d*_z_ ~ 3.7 mm, *d*_y_ ~ 7.3 mm), as is the case for RES-loaded (see Fig. 6c) cells where the reconstructed diameter of the tube is *d*_z_ ~ 8.2 mm and in y-plane *d*_y_ ~ 11.2 mm. Accordingly, the extraction of the precise location and shape of the tube contain large uncertainties for RES-loaded cells. This is more reliable for the other two systems (SynP50, SynC30). These imaging experiments confirm the analysis results of the two-voxel analysis from 3.2. Again, it is evident that the reconstructed cell bolus is notably compact when SynP50-loaded cells are considered, displaying an almost Gaussian distribution within the tube. However, in the experiment involving SynC30-loaded cells, the cell bolus was intermittently disrupted by air bubbles, resulting in isolated hotspots, each containing a few tens of thousands of cells. The achievable high resolution of SynC30-loaded cells enabled for imaging these features by MPI. In the case of RES-loaded cells, the resolution was insufficient for precise sample localization within the FoV. Nonetheless, tracking the signal maximum accurately determined the tube's position within the FoV even for this system.

**Figure 7:**
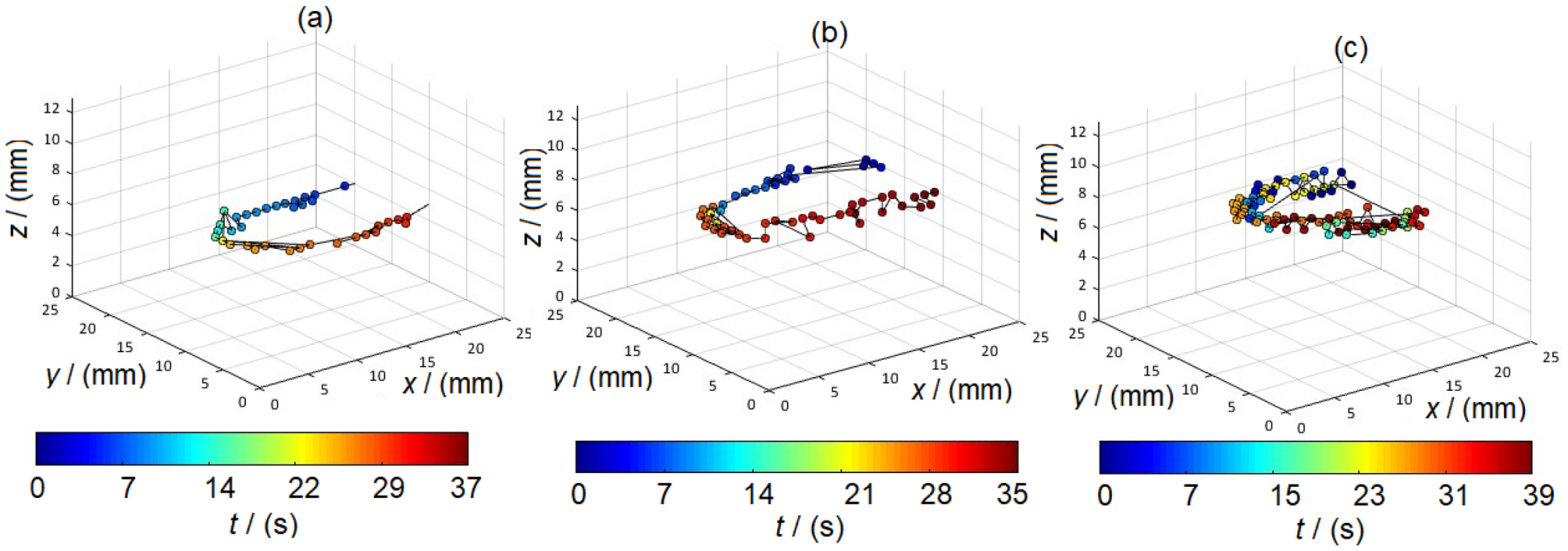
Signal maximum of the cell bolus for (**a**) SynP50, (**b**) SynC30 and (**c**) RES determined and tracked at each point in time.

Figure 7 illustrates the temporal and spatial cell hot spots by visualizing the voxel-by-voxel trace of the signal maximum from MNP-loaded cells at each measurement time point, as indicated by the color coding.

It can be seen that the localization of the bolus maximum of SynP50-loaded cells reflects the shape of the tube phantom very well. In contrast, the localization of the bolus with SynC30-loaded cells appears somewhat less accurate, although it should be noted that only one of the detected hotspots with a significantly lower iron mass was tracked. RES-loaded cells show a large scattering of signal maxima, which is due to the poor MPI performance of the RES-loaded cells. Nevertheless, it can be stated that localization and spatial tracking of the bolus was possible for all three samples and corresponded to the position of the tube in the FoV.

## Summary and conclusion

In this study we demonstrate for the first time the quantitative real-time tracking of MNP-loaded cells by MPI in a controlled phantom environment. Our experiments highlight the significant impact of MNP type on MPI resolution and imaging results. The choice of MNP and its associated characteristics lead to variations in the cellular uptake behavior, MPI signal quality, dispersion properties of the cell pellet and finally, the quality of cell bolus imaging. The best results in this regard were achieved with SynC30-loaded cells. The particles were completely internalized by THP-1 monocytes and surprisingly produced a better MPI signal compared to the initial state. We found that real-time MPI imaging allowed for the complete retrieval of the injected cell amount, which interestingly was also possible for cell samples with lower MPI image quality. For SynC30-loaded cells, a quantitative image analysis was possible from a quantity of 83 cells per voxel. This result is in the same order of magnitude as calculated in other studies^28^ With these findings, quantitative real-time MPI of MNP-loaded cells becomes possible. The remarkable temporal resolution of the MPI technology enables real-time tracking over a field of view of 19.2 × 19.2 × 9.6 mm^3^, making it ideal for preclinical imaging and the study of cell behavior in various contexts. Nevertheless, the initial experimental conditions were tailored to enable precise cell tracking under controlled settings. Subsequently, in advancing the feasibility study, it is imperative to employ physiologically relevant media like whole blood. Additionally, utilizing physiological vessel geometries, such as those attainable through 3D printing, can effectively serve as phantoms.

These findings underscore the importance of selecting the appropriate MNP type for specific applications and highlight the potential of MPI as a tool for quantitative, real-time cell tracking and imaging. In the future, the inflammatory cells used here are intended for MPI detection of pathological processes.

### Supplementary Information

Supplementary Videos.

Supplementary Figure 1.

Supplementary Figure 2.

Supplementary Videos.

## Data Availability

All data generated or analyzed as part of this study are included in this published article and its supplementary information files.
