# Supplementary material for: Counting cells in motion by quantitative real-time magnetic particle imaging
Source: Sci Rep. 2024 Feb 21;14:4253. doi: 10.1038/s41598-024-54784-5 (PMC10879211; doi:10.1038/s41598-024-54784-5)
Supplement: Supplementary file 2 — Supplementary Figure 1. [file 41598_2024_54784_MOESM2_ESM.docx]

# Appendix


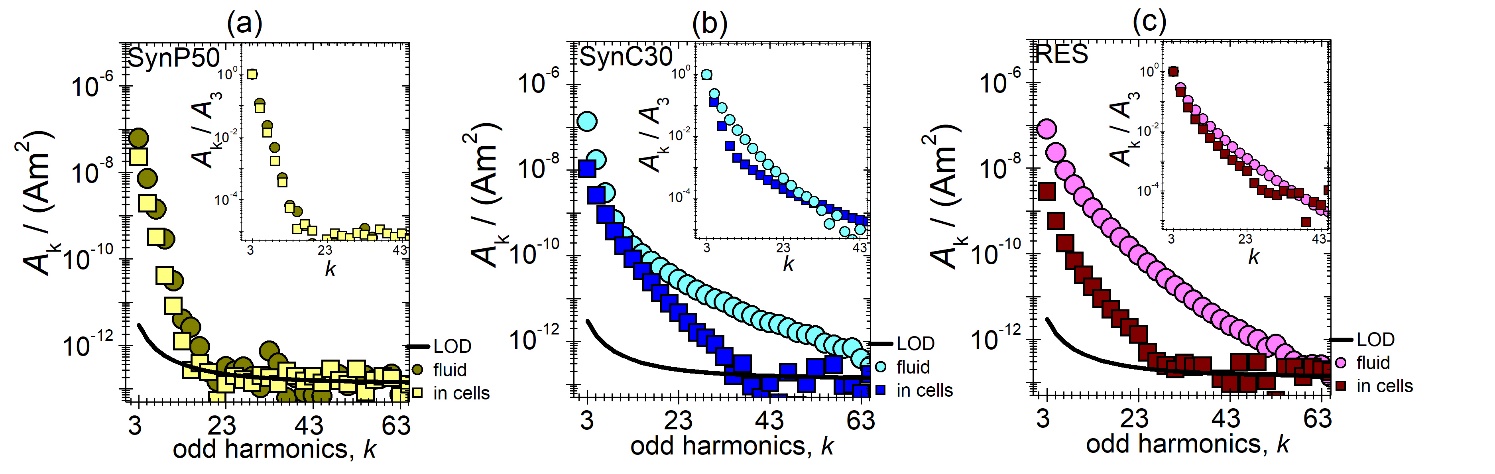


*Figure A.1: Amplitude spectra A_k_ of SynP50 (a), SynC30 (b), and RES (c) in initial state (circles) as well as after cellular uptake (10 min incubation, exposed iron mass of 28 µg) and washing of cells (squares). The insets display normalized amplitude spectra A_k_/A_3_, emphasizing the spectrum's shape variation.* *The black line denotes the LOD (mean + 3 SDV) of ten background measurements without any magnetic material*.
