# Supplementary material for: Counting cells in motion by quantitative real-time magnetic particle imaging
Source: Sci Rep. 2024 Feb 21;14:4253. doi: 10.1038/s41598-024-54784-5 (PMC10879211; doi:10.1038/s41598-024-54784-5)
Supplement: Supplementary file 3 — Supplementary Figure 2. [file 41598_2024_54784_MOESM3_ESM.docx]

# Appendix


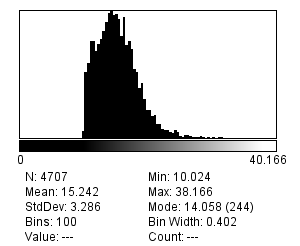

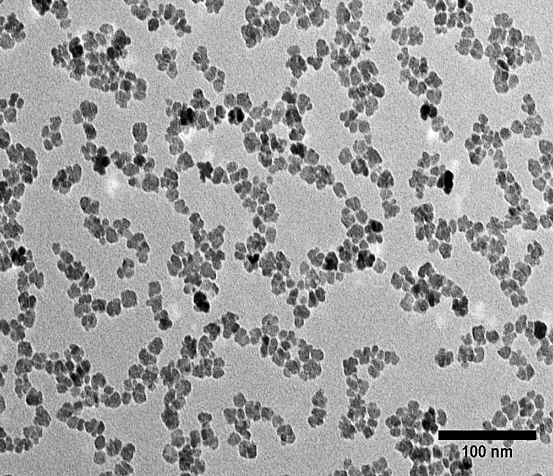
a)


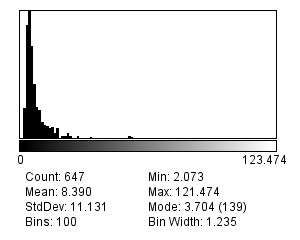



b)

Figure A.2: Transmission Electron Microscopy (TEM) images with histogram of a) Sync30 and b) RES.
